# How three-dimensional sketching environments affect spatial thinking: A functional magnetic resonance imaging study of virtual reality

**DOI:** 10.1371/journal.pone.0294451

**Published:** 2024-03-11

**Authors:** Yu-Hsin Tung, Chun-Yen Chang

**Affiliations:** Department of Horticulture and Landscape Architecture, National Taiwan University, Taipei, Taiwan; Georgetown University Medical Center, UNITED STATES

## Abstract

Designers rely on sketching to visualize and refine their initial ideas, and virtual reality (VR) tools now facilitate sketching in immersive 3D environments. However, little research has been conducted on the differences in the visual and spatial processes involved in 3D versus 2D sketching and their effects on cognition. This study investigated potential differences in spatial and visual functions related to the use of 3D versus 2D sketching media by analyzing functional magnetic resonance imaging (fMRI) data. We recruited 20 healthy, right-handed students from the Department of Horticulture and Landscape Architecture with at least three years of experience in freehand landscape drawing. Using an Oculus Quest VR headset controller and a 12.9-inch iPad Pro with an Apple Pencil, we tested participants individually with 3D and 2D sketching, respectively. When comparing 2D and 3D sketches, our fMRI results revealed significant differences in the activation of several brain regions, including the right middle temporal gyrus, both sides of the parietal lobe, and the left middle occipital gyrus. We also compared different sketching conditions, such as lines, geometrical objects (cube), and naturalistic objects (perspective view of a tree), and found significant differences in the spatial and visual recognition of brain areas that support visual recognition, composition, and spatial perception. This finding suggests that 3D sketching environments, such as VR, may activate more visual–spatial functions during sketching compared to 2D environments. The result highlights the potential of immersive sketching environments for design-related processes and spatial thinking.

## Introduction

Sketching is an important part of the design thinking process, as it allows designers to visualize and experiment with their ideas. As Aristotle once wrote, “the soul never thinks without a mental image” (p. 177) [1]. He illustrates how the visualization of an image is critical to thinking. For landscape designers, sketching is an essential way to represent an image conceived in the mind, which is a critical means of constructing arguments for the design task [2]. Visualization through sketching helps to improve the creation and manipulation of space and form and conveys the conversation of imagination [3]. Through sketching, designers envisage better solutions and possibilities with more precise solution than with creative thinking alone [4, 5]. As technology has developed, many tools now aid designers in visualizing their sketches. In terms of sketching media, the tools can be broadly classified as two-dimensional (2D) tools (e.g., pencil-and-paper and graphics tablets) and three-dimensional (3D) tools, such as computer modeling and VR (e.g., computer-aided design systems and gesture-based interfaces) [6]. Wu et al. [7] found that digital media has made it possible for designers to produce in-depth descriptions of detailed designs with the help of related functions in digital media, while the traditional pen-and-paper environment allows designers to use synthetic and contrasting methods to manipulate their idea development.

As a type of sketching media, VR has recently become increasingly popular in many research fields, including environmental simulation, technical operations, and design education [8–10]. VR refers to a computer-generated experience that creates a sense of immersion and presence [11]. It is a powerful tool for increasing design concept generation [12] and operation training performance [13], and it is widely used in design review visualization media [14]. The efficiency of VR sketching media for practicing spatial ability has prompted their increased use in environmental simulations, technical operations, and design-related educational practices [8–10]. According to the degree of immersion, VR systems can be assigned to three categories: *nonimmersive* (desktop display), *semi-immersive* (screen-projected display), and *immersive* (head-mounted gesture display) [15]. Gesture-based immersive VR interfaces permit 3D environments to create the full virtual sensation of interacting on a real-world scale through headsets and remote controllers [16, 17]. A recent study by [18] found that immersive VR head-mounted displays are more suitable than 2D screens for training 3D movements because they are more motivating to user and easy to operate. Paes et al. [19] also found that immersive VR systems provide users with an enhanced 3D perception of virtual models and greater levels of presence compared to nonimmersive systems, which can benefit collaborative design reviews and increase productivity. These articles suggest that immersive VR tools can enhance users’ perceptions and understanding of a 3D space while consuming fewer cognitive resources during mental imaging (e.g., molecular structure, materials, and components) [15–17, 20–22]. Furthermore, VR tool is considered to provide higher experimental control over stimuli, with validity as an investigational medium for capturing brain activation profiles [23]. This experience of immersion, interaction, and involvement supports enhancing the perception and understanding of the 3D space [24, 25].

Research by [26] found that visualization environments that better match human perceptual and interaction capabilities to the task at hand improve our understanding of 3D visualizations. Similarly, [27] showed that using an immersive virtual environment in a design studio can enhance spatial perception, improve designs, and affect the design process by changing an individual’s method of thinking in architectural design. Therefore, 3D sketching tools, such as VR, have great potential for practicing spatial and design thinking in the realm of spatial visualization [9, 10]. A distinguished example is “Foldit” [28], an online game that allows players to design stable protein structures to advance their understanding of protein folding and prediction. 3D visualization is essential to making this process more accessible compared to traditional methods, which are extremely difficult and time-consuming.

However, most studies in design-related fields have primarily focused on comparing the outcomes of practices in 2D- and 3D-based sketching environments, a vital question remained unanswered in the brain activation caused by using different sketching tools. In empirical research, the mechanism through which viewing sketches in a 3D virtual environment affects the brain has been less studied. The current study investigated the differences in brain activation observed when participants viewed 2D and 3D environmental sketches. The study did not consider movement or the ability to sketch but instead focused on the spatial processes involved in viewing different sketching tools and sketching tasks that affect the visual and spatial functions of the brain. This research aims to investigate the neural activation involved in viewing the spatial sketching process and to distinguish the effects of brain activation caused by 2D and 3D visual sketching environments.

### The role of spatial processing in sketching and its neural correlates

This section discusses the importance of spatial representation in sketching and highlights the differences between 2D and 3D sketching environments. Sketching involves precise perceptual processing, especially in a 3D spatial context in which locations and objects play a role in spatial representation. Viewing a sketch involves a complex set of brain processes connected to perceiving, processing, and interpreting visual information. Sketching with a 3D virtual tool permits users to create higher-fidelity visualizations of spatial depth and space navigation than sketching with a 2D tool [29]. When sketching using a 2D tool, our perception transforms and manipulates the image into a 3D image; the representation of these characteristics depends not only on the objects’ arrangement, orientation, and perspective scale but also on the information conveyed by 2D and 3D spatial transformation views. In contrast, when sketching with a 3D tool, the visualization of the depth and navigation provided by the computer helps the user visualize the object as a whole [30–32].

**Fig 1:**
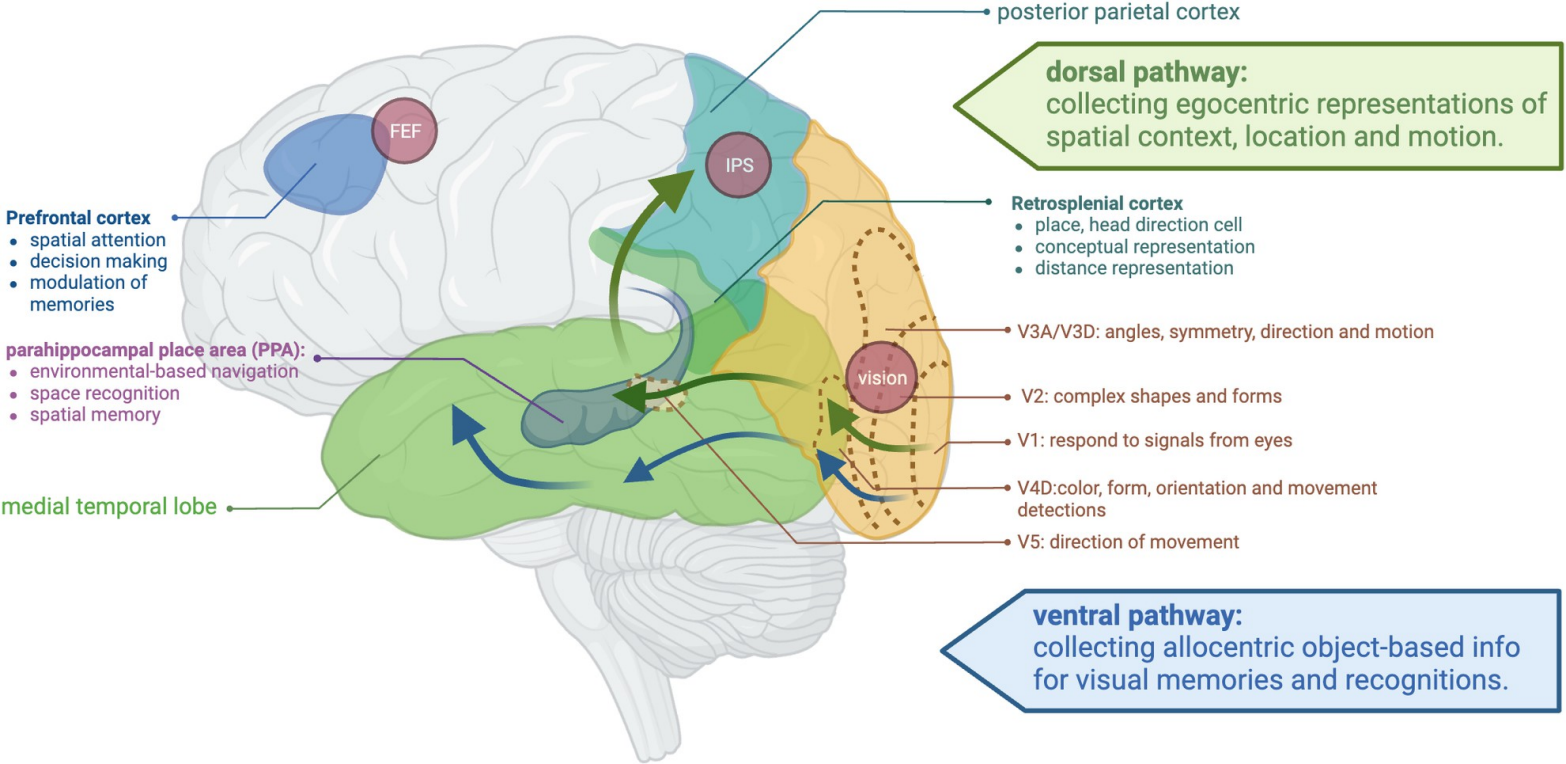
Visual and spatial information processes and functions. Illustration publication number: WW2620UWAC.

Spatial processing refers to the cognitive functions of perceiving, analyzing, and manipulating information about the spatial relationships between objects and locations in the environment; these processes are symmetrically distributed in both hemispheres [33]. During spatial processing, the brain achieves spatial representation through two distinct neural pathways: the dorsal and ventral pathways. The dorsal pathway, also known as the “where” pathway, is responsible for processing information related to the location and movement of objects in the environment. This pathway is involved in forming egocentric representations of spatial context, spatial navigation, and motor planning. In contrast, the ventral pathway, also known as the “what” pathway, is responsible for processing information related to the identity and properties of objects in the environment. This pathway is involved in forming allocentric representations of spatial context, object recognition, and categorization. Both dorsal and ventral pathways are interconnected and work together to facilitate a complete understanding of visual information [33–36]. The dorsal pathway is primarily associated with the posterior parietal cortex and the medial temporal lobe, while the ventral pathway is associated with the inferior temporal cortex and the occipital lobe [33–35, 37] (Fig 1). The following section explores the role of attention, navigation, and imagery in the process of viewing sketches.

### The role of attention, navigation, and imagery in viewing sketches

While the core spatial processing network is symmetrically distributed in the dorsal frontal-parietal regions (presupplementary motor area), the anterior insula and frontal operculum of both hemispheres suggest that attention, navigation, and imagery play important roles in sketching [33, 38]. The dorsal frontal-parietal regions are consistently implicated in spatial processing across a range of tasks [39]. The dorsal parietal cortex mediates top-down visual attention and the selection of stimuli, while the ventral frontoparietal network regulates the bottom-up orientation of attention and the task-relevant stimuli of visual perception [40]. The frontal-parietal areas, including both the frontal and parietal lobes, are bilaterally involved in a wide range of cognitive processes, such as attention, working memory, decision-making, and motor control-related spatial information [38]. The parietal cortex is responsible for visual–spatial attention in spatial perception, and in planning and executing movements, which helps designers focus on specific parts of the sketch. Meanwhile, the prefrontal cortex is responsible for interpreting the sketch and making sense of it. On the other hand, the ventral frontoparietal network is responsible for processing object recognition and perception. This network is closely connected with the inferior parietal lobe, which plays a crucial role in visual–spatial processing.

To delve deeper into the underlying mechanisms, it is crucial to consider the role of spatial navigation. Spatial navigation plays a critical role in spatial processing and is closely related to long-term memory. Recent single-cell investigations have identified neurons that regulate and encode spatial properties during navigation, including place cells, grid cells, and head direction cells [41, 42]. Studies have shown that the parahippocampal place area (PPA) is activated when individuals view or imagine scenes or environments. In particular, the PPA is involved in the recognition and processing of spatial information, such as the layout and organization of objects within a scene. This makes the PPA important for visual–spatial thinking and sketching, as it helps an individual to mentally visualize and manipulate spatial information.

While PPA is responsible for recognizing specific views in environment-based navigation, the retrosplenial cortex (RSC) converts allocentric to egocentric representations [43]. The ability to generate mental imagery is critical for sketching, and the RSC is thought to play a role in mental imagery and the construction of spatial maps [44, 45]. Studies have shown that this region is activated when individuals are asked to imagine and mentally manipulate objects in space or navigate through virtual environments. Thus, the RSC is an important component of the brain’s network for spatial cognition and is involved in a range of processes that contribute to visual–spatial thinking and sketching.

As we delve further into cognitive processes, spatial memory emerges as a key player. Spatial memory refers to the ability to remember and mentally manipulate spatial information, which is supported by brain regions, such as the hippocampus [46]. Research have shown that sketching influence the demand for spatial memory in interpreting or remembering specific aspects of design [47]. While hippocampus and other related brain regions are involved in recalling and navigating the physical environment [48]. The prefrontal and anterior cingulate cortex are critical for the storage and retrieval of remote spatial memories involving parietal and RSCs during the consolidation of remote memory [49].

The right hippocampus appears to be particularly involved in memory for locations within an environment, with the left hippocampus being more involved in context-dependent episodic or autobiographical memory [50]. Robin et al. [51] explored how spatial context plays a crucial role in the neural representation of events with functional magnetic resonance imaging (fMRI) and showed that the hippocampus and surrounding areas were more active when events were presented in a spatial context, suggesting that spatial information is prioritized in the neural representation of events. The use of VR on spatial memory is also related to the method of loci by which one links their spatial scenario with objects to increase memory capacity [52].

### Spatial ability is influenced by individual factors

Individual factors, such as age, gender, and background, can influence the spatial cognition involved in sketching [53]. Studies have shown that spatial ability tends to decline with age and that there is a gender difference, with men typically outperforming women in spatial tasks. Additionally, research has found that users with different spatial abilities exhibit distinct drawing behaviors in VR, and that spatial ability can impact the shape of the drawing [54]. A person’s spatial ability can be assessed using various methods [55, 56]. Pellegrino (1984) proposed that spatial ability can be broken down into two major factors: spatial relations and spatial visualization ability [57]. The Purdue spatial visualization test (PSVT) is commonly used to evaluate spatial ability, particularly the capacity to mentally rotate 3D objects [58]. The rotation test (PSVT:R) is a validated test for measuring intrinsic and static spatial abilities, including psychovisualization and mental image rotation [59, 60]. It consists of 30 items to be completed within 20 minutes and demonstrates high internal consistency according to the Kuder–Richardson formula. Researchers across various fields have used PSVT:R to measure individual spatial abilities. While, in this study, we did not consider spatial ability a major variable, it was considered a covariate variable.

## Materials and methods

This study compared the cognitive effects of using 2D and 3D sketching tools for different tasks. To observe brain activation related to viewing 3D sketching while also avoiding head motion, the experiment was split into two stages: *sketching-recording* and *viewing-scanning*. The participants first completed sketching tasks with both 2D and 3D tools in the sketching-recording. Then, during the viewing-scanning stage, which was conducted separately to avoid head motions, they viewed their sketches while their brain activity was recorded with fMRI. The results were then compared to determine whether there were any noticeable differences in brain activation either when viewing 2D and 3D sketching environments or between the different sketching tasks.

### Experimental design

All participants were asked to complete the sketching-recording stage using each setup sequentially, starting with the 2D tool and then moving on to the 3D tool. Prior to the start of the first stage, participants received comprehensive information about the study and provided their consent by signing a consent form.

The sketching-recording stage involved 1) a spatial task, 2) a tutorial exercise, and 3) six recording tasks. All participants were asked to complete the first stage before starting the second stage. The tutorial exercise lasted approximately 15 minutes and was designed to ensure that participants understood how to use both tools. During the tutorial exercise, the experimenter introduced the 2D tool with manual instructions and asked the participants to draw horizontal lines, a cube, and an object on the sketching board using a stylus. Printed images were provided to help clarify the criteria. Next, the participants were asked to use the VR device and the right-hand controller to draw horizontal lines, a cube, and an object perspective in the sketching space. Printed images were also provided to clarify the criteria. Participants were permitted to take as much time as they needed during the tutorial exercise to become familiar with both setups. All participants successfully completed the exercise within 15 minutes. Once the participants were familiar with both tools, the experiment began.

**Fig 2:**
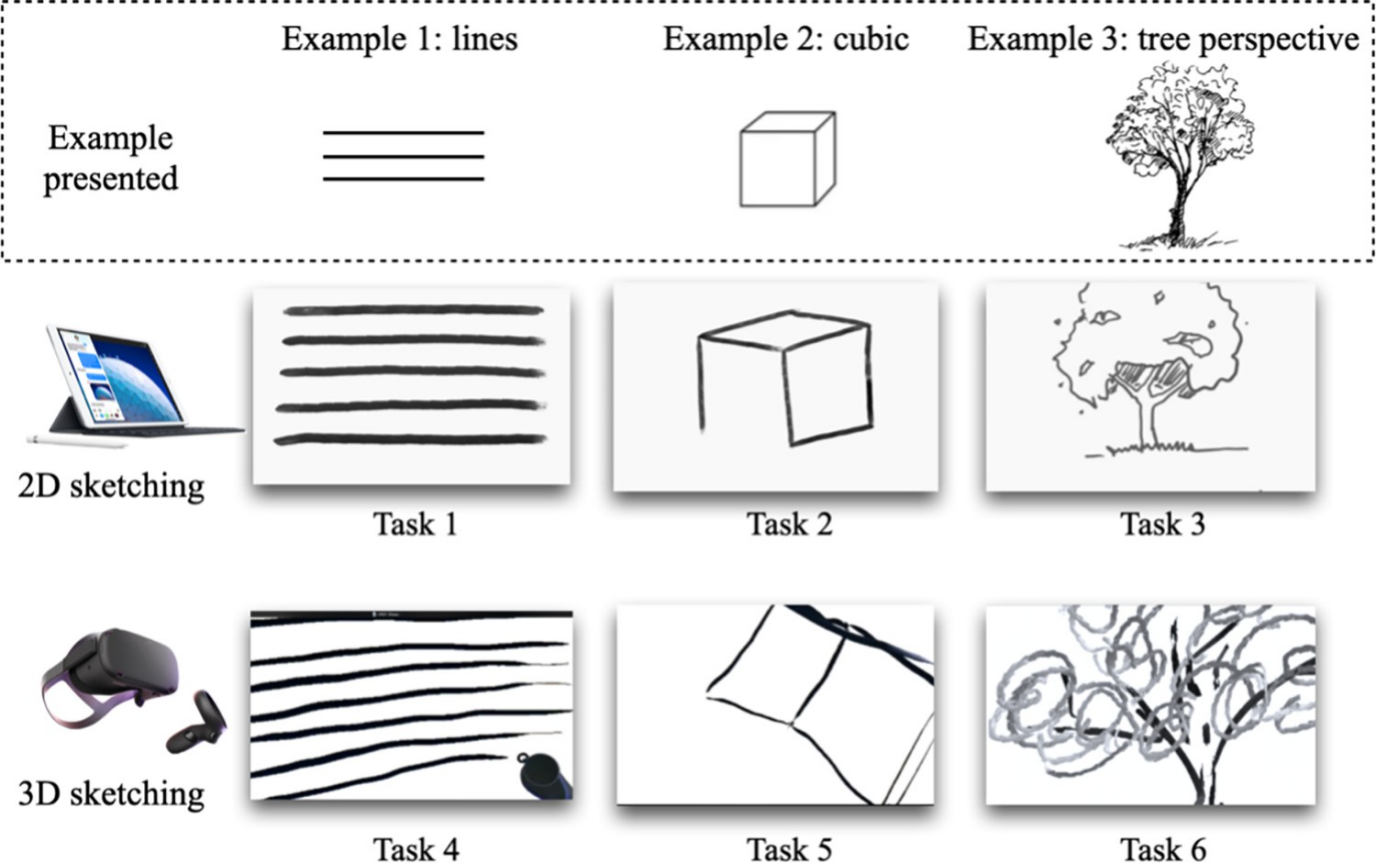
Sequential 2D and 3D sketching tasks: Lines, a geometrical object, and a naturalistic object.

All participants completed six tasks in sequence using the 2D and 3D tools provided in the experiment. The participants were asked to draw, using each tool, the following three elements: lines, a geometrical object (the cube), and a naturalistic object (a tree perspective). Starting with the 2D tool, the tasks were administered in a specific sequence, and participants performed the line task, followed by the geometrical object task and, finally, the naturalistic object task. Subsequently, participants proceeded to the 3D tool and performed the same tasks in the same order (see Fig 2). To ensure that the participants focused on the sketching process, the sketching time for each task was limited to 30 seconds. The 3D setup included a 2019 wireless head-mounted Oculus Quest VR display (128 GB and 1440 × 1600 pixels per eye at 72 Hz) and a virtual two-handed controller for use with the Tilt Brush 3D drawing application (Google Co., 2006). The 2D drawing setup included a 12.9-inch iPad Pro and Apple Pencil (2018) and the Adobe Photoshop application, which has a functional layout similar to Tilt Brush. Both setups were set to an empty sketching canvas with a white background, and the only available stroke color was black.

Following the sketching-recording stage, the experimental design progressed to the viewing-scanning stage. During this stage, the participants watched the video recordings of their sketching tasks while being monitored by a Siemens 3T MAGNETOM Prisma magnetic resonance imaging (MRI) platform at the Image Center for Integrated Body, Mind, and Culture Research at National Taiwan University. All six sketching videos completed during the first stage were viewed by the participants in the second stage.

**Fig 3:**
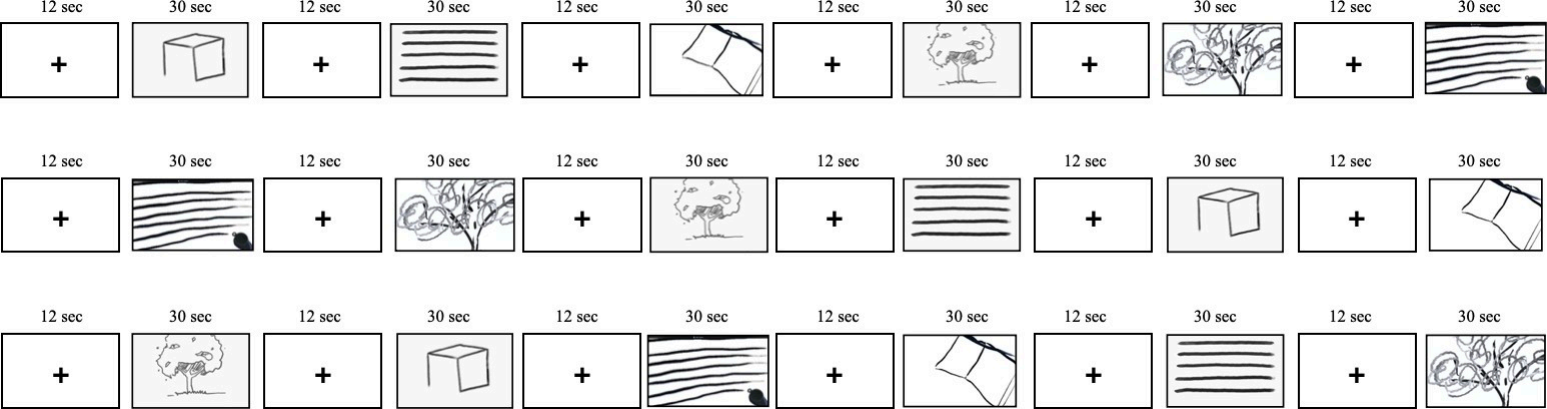
The fMRI viewing-scanning stage process in which participants watched prerecorded sketching videos in pseudorandom order.

We pseudorandomly assigned the order in which the participants viewed the videos in three repeated sequences to ensure robust responses. Each sequence included three tasks performed using both sketching tools, resulting in a total of 18 videos (3 tasks × 2 sketching tools × 3 times), as depicted in Fig 3. The tasks were presented using a block design with interleaved trials to avoid consecutive presentations of the same tool type. This approach maximized the statistical power and created a different spatial sketching experience for the participants.

### Participants and ethics

The study recruited 20 right-handed students (10 female) from the Department of Horticulture and Landscape Architecture (mean age = 24.4 years; SD = 2.23 years). All participants had normal or corrected-to-normal vision, at least three years of landscape sketching experience, and formal training by the university. None of the participants claimed a history of psychological, physical, or neural symptoms or injury.

The Behavioral and Social Science Research Ethics Committee at National Taiwan University approved the study, and all participants provided informed consent by signing the consent form (Ethical Review Approval No.: 201908HM003). Each participant received NT$500 (approximately USD$17) as monetary compensation for their participation in the study.

### Spatial ability tasks as cognitive controls

Spatial ability is the capacity to mentally represent, manipulate, and comprehend the relationship between object positions within a visual stimuli [55, 56]. To address the potential confounding factor of spatial ability, this study utilized PSVT, as recommended by previous studies [59, 60]. Data were also gathered to test whether gender and age affected spatial ability. The study examined the effects of age and gender on spatial ability by conducting a two-way analysis of variance (ANOVA), which considered the impact of both independent variables and their interactions on spatial ability scores. The results of the analysis are presented in Table 1 and reveal no significant interaction between age, sex, and spatial ability scores, which indicates that the effects of these variables on the dependent variable were not interdependent (see Table 1).

**Table 1:** Interaction between gender and age on spatial ability.

Source of variation	SS	*df*	MS	*F*	p-value	Eta value
age	80.51	7	11.5	0.79	0.616	0.442
gender	0.31	1	0.31	0.02	0.888	0.003
interaction	13.68	4	3.42	0.24	0.909	0.119
error	101.5	7	14.5			
total	4700	20				

We further examined the main effect of gender and age on spatial ability, and the results showed no significant difference in spatial ability test scores between males (mean [SD], 15.4 [2.83]) and females (mean [SD], 14.6 [3.72]); *t*_(18)_ = 0.54; p = 0.59; *d* = 0.24 (see Table 2) or based on age (F_(5,12)_ = 1.256, p = 0.347) (see Table 3).

**Table 2:** Spatial scores for the independent sample t-test according to biological sex.

	Mean		*df*	*t*	p	Cohen’s *d*
	Male (n = 10)	Female (n = 10)				
Spatial task	15.4	14.6	18	0.541	0.595	0.242

**Table 3:** One-way ANOVA results for the spatial ability scores between age levels.

Predictors	SS	*df*	MS	F	p	Eta value
age	84.583	7	12.083	1.256	0.347	0.423
error	115.417	12	9.618			
sum	200	19				

### MRI setup acquisition and data analysis

The recorded stimuli videos were displayed on the screen of an MR-compatible image interaction platform using EPrime 2.0 software. MR images were acquired using a Siemens 3T MAGNETOM Prisma MRI scanner with a 32-channel head coil.

The T1-weighted high-resolution images were acquired at the following settings: TE(echo time) = 2.3 ms, TR(repetition time) = 2000 ms, FA = 8°, matrix size = 256 × 256 × 192, FOV(field-of-view) = 240 × 240 mm^2^, voxel size = 0.93 × 0.93 × 0.93 mm^3^, a bandwidth of 200 Hz/pixel, and number 192 slices. We obtained the T2-weighted images at the following settings: TE = 103 ms, TR = 953 ms, FA = 150°, matrix size = 288 × 384 × 045, FOV = 192 × 192 mm^2^, voxel size = 0.66 × 0.50 × 3.00 mm^3^, and number of slices = 45. This was followed by the 2D echo-planar image sequences acquired through 252 scans, with TE = 30 ms, TR = 3000 ms, FA = 90°, matrix size = 64 × 64 × 45, FOV = 192 × 192 mm^2^, voxel size = 3.00 × 3.00 × 3.00 mm^3^, and a bandwidth of 2440 Hz/pixel (interleaved slice order/ bottom up).

#### Preprocessing

All the Digital Imaging and Communications in Medicine (DICOM) images were first reconstructed into the NIfTI-1 format (.nii) using *DICOM import* in Statistical Parametric Mapping tool version 12 (SPM12) [61]. Subsequently, we performed the following preprocessing procedure. First, we conducted motion correction (realign and unwarp) to account for motion artifacts in the fMRI time series. This involved realigning all Echo Planar Imaging (EPI) scans (each with 252 slices) to correct for translational and rotational motion for each participant. We used a Gaussian smoothing kernel with a full width at half maximum (FWHM) of 5 mm. The images were then resliced to match the mean of the image. No wrapping or weighting was applied. We checked the translation and rotation of the *x*, *y*, and *z* axes of the head center for each participant. According to the SPM list, the acceptable translation and rotation ranges are both within ± 5 mm. For the present experiment, translation and rotation within the *x*, *y*, and *z* axes were checked, with acceptable ranges set at ± 3 mm. Second, we conducted slice timing correction to account for differences in image acquisition time between slices. The fMRI data consisted of 45 slices per volume with TR = 3000 ms. The reference slice was set as the middle slice (23), and the slice order was bottom up and interleaved.

Third, we coregistered the high-resolution anatomical image to the functional images using coregistration algorithms. This step improved the alignment of images with the Montreal Neurological Institute (MNI) template. We used the mean EPI image as the reference image and the T2-weighted image as the source image, as they shared the same slice sequence and number. The objective function was set as normalized mutual information with default separation and tolerances according to SPM 12 settings.

Fourth, to enhance the interpretation of the brain images, we applied segmentation to separate the images into cerebrospinal fluid, white matter, and gray matter for easier interpretation. We saved bias-corrected images to ensure uniform intensities within different tissue types. We affined regularization to the (International Consortium for Brain Mapping (ICBM) space template of East Asian brains for local optimization, and forward deformation was used to normalize the images to the MNI space.

Fifth, we performed normalization, and a more accurate analysis of individual brain images was modified into brain templates (bounding box = [−78 −112 −70 78 76 85]) with a default voxel size [3 3 3]. Finally, spatial smoothing was applied to improve the signal-to-noise ratio. We used a filter kernel with an FWHM of [6, 6, 6], which is twice the voxel size, effectively strengthening the signal and reducing noise.

#### fMRI data analysis

To examine the brain scans of individual participants while they viewed various sketches, we conducted a first-level analysis. This analysis employed a t-test using the SPM Menu protocol and considered six distinct conditions. Each condition involved a specific video block design lasting 30 seconds, interspersed with resting intervals of 12 seconds. Conditions 1–3 used 2D tools for sketching lines, geometric objects, and natural objects, respectively. Conditions 4–6 employed 3D tools for sketching lines, geometric objects, and natural objects, respectively. These conditions were presented in a pseudorandom sequence within a single session. The general linear model (GLM) was applied to measure the blood oxygenation level dependent (BOLD) signal and estimation. The initiation of each block was determined at a precise time point as follows: Condition 1 = [172 s, 424 s, 718 s], Condition 2 = [4 s, 298 s, 592 s], Condition 3 = [130 s, 256 s, 676 s], Condition 4 = [46 s, 382 s, 508 s], Condition 5 = [88 s, 466 s, 550 s], and Condition 6 = [214 s, 340 s, 634 s]. The realignment parameter was also applied as a regressor to eliminate head motion potential. No predefined masks were chosen at the p-value of FWE = 0.05 at the extent threshold of > 39 voxels according to the SPM cluster size threshold calculation [62]. The single-subject results were visualized using the xjView toolbox (https://www.alivelearn.net/xjview).

For the group statistical inference, we conducted groupwise analysis using the SPM tool to specify the second-level analysis with the 2 × 3 flexible factorial design [2 (2D and 3D) × 3 (line, geometrical object, naturalistic object)] as coded (A1 = non-VR, A2 = VR, B1 = line, B2 = cubic, B3 = object). The main effects compared the non-VR (2D) and VR (3D) conditions with the consideration of spatial ability as a covariate variable. Furthermore, we examined the interactions among different tools across three conditions, enhancing our comprehension of how independent variables relate to differences in brain activation.

## Results

The goal of the experiment was to assess whether there were noticeable differences in brain activation when participants viewed 2D and 3D sketching environments. Prior to this, we conducted an initial check to confirm that the participants’ spatial abilities were consistent across age and gender groups. Furthermore, we categorized the sketching scenarios into three distinct tasks. The results of our analysis unequivocally revealed substantial differences in neural activation among participants when they observed 2D sketching processes in contrast to 3D sketching processes. Additionally, we conducted further comparisons between 2D and 3D sketching environments for tasks involving line drawing, geometric objects, and naturalistic objects. These comparisons also highlighted significant differences in brain activation, which will be elaborated on in the subsequent section.

### Comparison of 2D and 3D sketching environments

This section compares the participants’ brain activation patterns during their exposure to the sketching videos in both 2D and 3D environments. It examines any noteworthy differences in brain activity between these two settings to provide a comprehensive overview of the data analysis across all trials. As shown in Table 4, when comparing the views of the 2D and 3D sketching processes, significant differences were found on the right middle temporal gyrus (MTG), left middle occipital gyrus (MOG), left postcentral gyrus, and right subgyral (PFWE < 0.05, *T* = 5.07).

**Table 4:** Significant variations in brain activation observed across different areas when comparing 2D and 3D sketch viewing.

Laterality	Brain structure	MNI coordinate [*x*, *y*, *z*]	*t*-value	P_FWE-cor_	Cluster size
R	Middle temporal gyrus	42, −64, 5	18.99	< 0.001	1163
L	Middle occipital gyrus	−42, −70, 5	13.26	< 0.001	1228
L	Postcentral gyrus	−30, −37, 59	8.89	< 0.001	158
R	Parietal lobe subgyral	15, −52, 62	7.15	< 0.001	87

Height threshold: *T* = 5.07, p = 0.000 (0.050)

Extent threshold: *k* = 39 voxels, p = 0.000 (0.000)

*df*: 95

**Fig 4:**
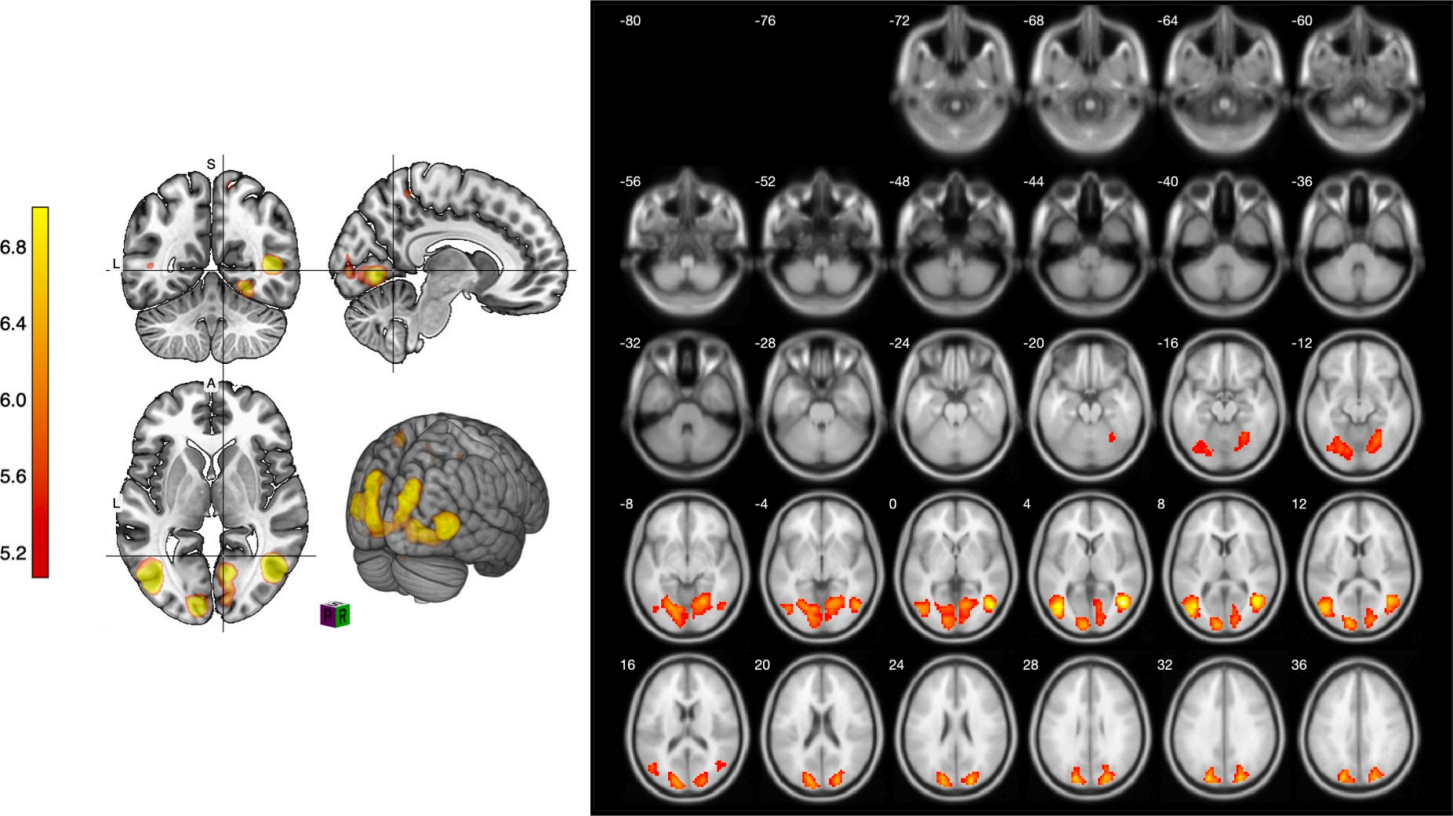
Slice images of comparison of viewing sketches in 2D and 3D environments.

Fig 4 shows the differences observed in cognitive function and brain activation when participants viewed the 3D sketching environment. These results support our research hypothesis that 3D sketching media stimulate different activations of brain activity compared to 2D sketching media.

### Viewing line sketches in 2D and 3D environments

**Fig 5:**
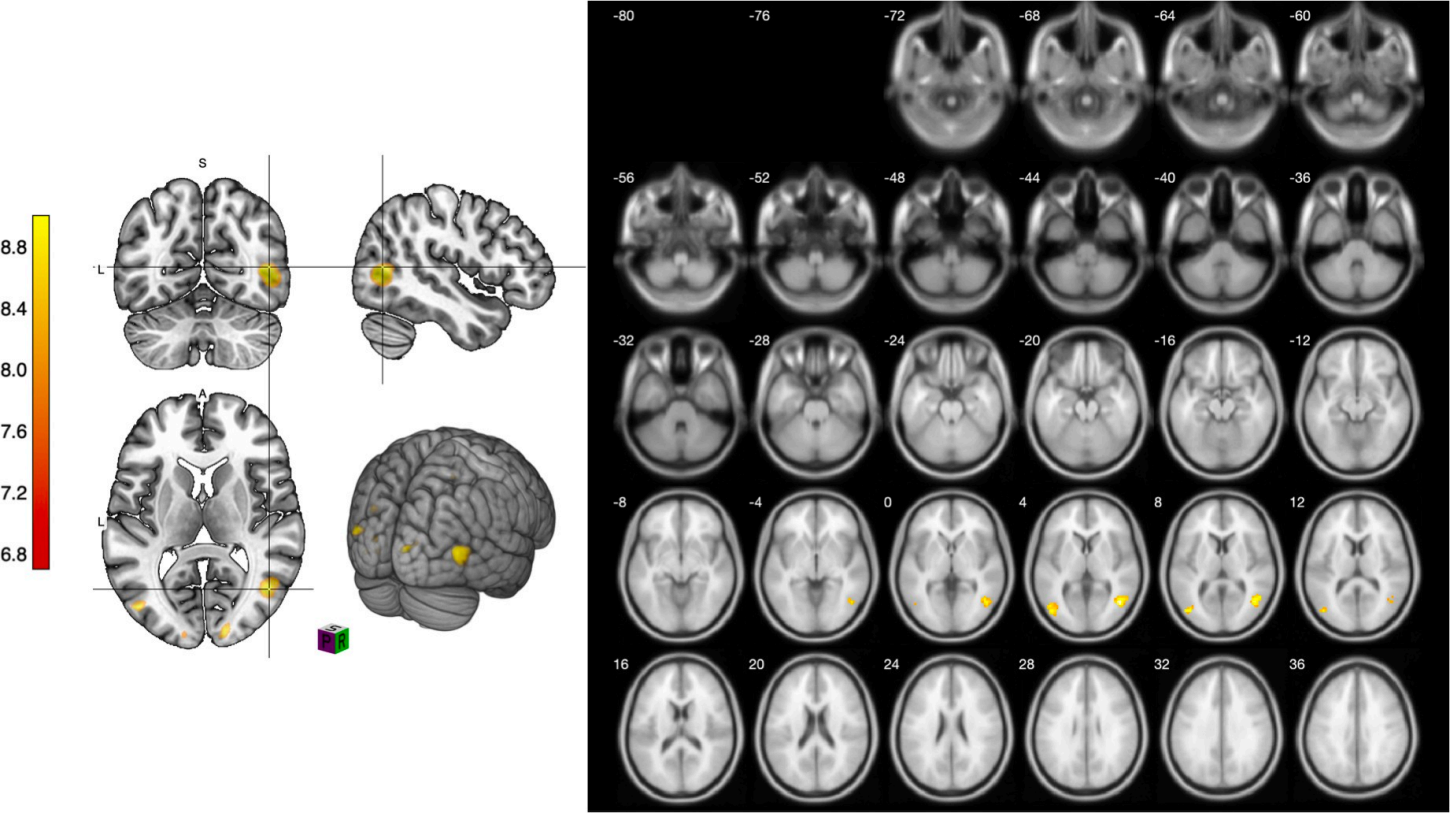
The slice images revealed significant activation in viewing the line sketching process when comparing the 2D and 3D environments.

We further focused on examining brain activation differences during the viewing of the line sketches in both 2D and 3D environments, investigating any distinctive findings. First, the results of the line sketching tasks (comparing Condition 1 to Condition 4) showed significant activation of the right MTG and the left MOG when viewing the 3D sketching environment (Table 5 and Fig 5).

**Table 5:** Results of the ANOVA comparing line conditions in the 2D and 3D sketching environments.

Laterality	Brain structure	MNI coordinate *x*, *y*, *z* (mm)	*t*-value	P_FWE-cor_	Cluster size
R	Middle temporal gyrus	45, −64, 5	13.22	< 0.001	81
L	Middle occipital gyrus	−45, −76, 5	10.18	< 0.001	43

Height threshold: *T* = 6.84, p = 0.000 (0.050)

Extent threshold: *k* = 39 voxels, p = 0.000 (0.000)

*df*: 19

### Viewing geometrical object sketches in 2D and 3D environments

**Fig 6:**
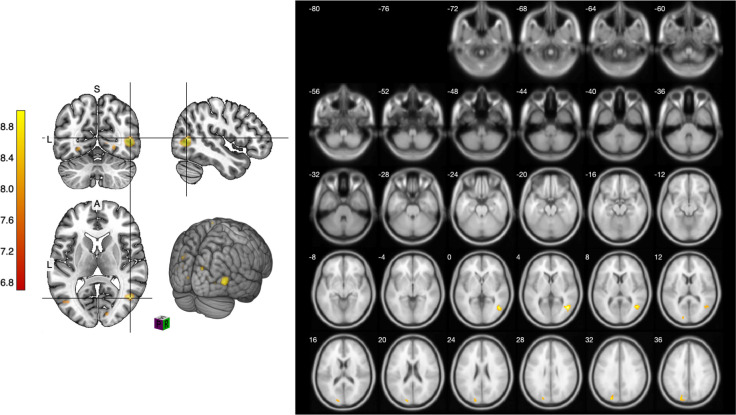
Slice images revealing areas of significant brain activation during the viewing of cube sketches, comparing 2D and 3D environments.

While observing the sketching process of geometric objects in both 2D and 3D environments, significant activations were identified in the right MTG and the left cuneus (Table 6). These activations were particularly pronounced when comparing the cube sketching process between the 2D and 3D environments (PFWE < 0.05, *T* = 6.74) (Fig 6).

**Table 6:** Brain region differences when viewing the cube sketch: 2D vs 3D environment.

Laterality	Brain structure	MNI coordinate *x*, *y*, *z* (mm)	*t*-value	P-_FWE-cor_	Cluster size
R	Middle temporal gyrus	45, −64, 5	13.54	< 0.001	73
L	Cuneus	−21, −85, 32	9.06	< 0.001	49

Height threshold: *T* = 6.74, p = 0.000 (0.050)

Extent threshold: *k* = 39 voxels, p = 0.000 (0.000)

*df*: 19

### Viewing naturalistic object sketches in 2D and 3D environments

**Fig 7:**
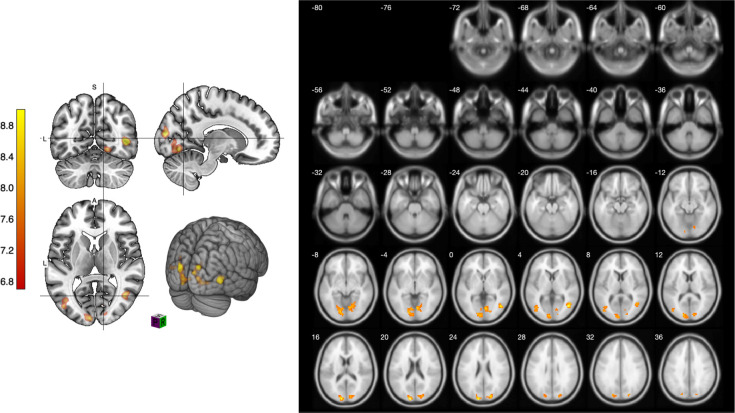
Slice images comparing object sketching processes viewed in 2D and 3D environments.

Next, we turn our attention to the comparison of naturalistic object sketches in both 2D and 3D environments. As shown in Table 7, we observed noteworthy activations in the bilateral cuneus, the MOG, and the right side of the lingual gyrus (PFWE < 0.05, *T* = 6.74) in the 3D condition. This finding underscores a substantial difference in brain activations when participants view naturalistic object sketches in 2D versus 3D environments (see Fig 7).

**Table 7:** Visual and spatial brain response mechanisms: Viewing object sketch in 2D and 3D.

Laterality	Brain structure	MNI coordinate *x*, *y*, *z* (mm)	*t*-value	P_FWE_	Cluster size
L	Cuneus	−12, −94, 20	16.68	< 0.001	254
R	Middle occipital gyrus	45, −67, 2	14.59	< 0.001	62
R	Cuneus	21, −88, 26	11.54	< 0.001	96
R	Lingual gyrus	6, −73, −1	10.17	< 0.001	130
L	Middle occipital gyrus	−42, −70, 5	8.7	< 0.001	41

Height threshold: *T* = 6.74, p = 0.000 (0.050)

Extent threshold: *k* = 39 voxels, p = 0.000 (0.000)

*df*: 19

## Discussion and conclusions

In this study, we investigated the differences in brain activation patterns that occurred when the participants viewed sketching processes in both 2D and 3D environments across various sketching tasks. Our findings unveiled significant disparities in brain activity when participants experienced sketching in 3D versus 2D environments. The disparities were particularly apparent in regions such as the right MTG, left MOG, left postcentral gyrus, and right parietal lobe. These observations revealed the intricate interplay between visual, spatial, and motor brain activation associated with 3D sketching tools, specifically in the temporal and occipital gyrus regions. Furthermore, a deeper examination of different sketching conditions revealed nuanced variations in neural activation. Specifically, under the line condition, significant activations were observed in the right MTG and left MOG in the 3D environment as opposed to the lack of activation observed in these areas under the same condition in the 2D condition. Meanwhile, the geometrical condition and the naturalistic condition both yielded activations in the cuneus. These observations offer a more detailed understanding of how sketching with different content in a 3D sketching environment elicits different neural activations during visual and spatial processes as compared to 2D sketching environment.

The activation of the right MTG is known for its role in semantic memory processing, multimodal sensory integration, and spatial processing [63]. Furthermore, research has shown that MTG is necessary for forming nonsalient, novel, and creative insights [64]. Our findings suggest that viewing linear and geometrical object sketches in 3D environments may involve similar brain activation, while 3D freehand sketching is less related to accuracy than to the relationships between shapes and space.

The MOG is involved in early visual processing and is associated with basic visual perception [65]. During the line condition and naturalistic object condition, but not in the geometrical object condition, the MOG played a critical role when the participants viewed the sketch in a 3D environment. While the MOG was more pronounced when viewing natural objects in 3D than when viewing them in 2D, it was still involved significantly in the spatial tasks, such as sketching in the virtual space. Furthermore, the cuneus and the lingual gyrus, which are involved in basic visual–spatial processing [66], were also critical areas in 3D object sketching but not in 3D line sketching. While the cuneus is known to receive basic visual–spatial information from the retina [67], our study showed that the 3D sketching environment of both geometrical and naturalistic objects demands higher visual–spatial cognition.

The parietal lobe is crucial for spatial tracking [68], depth perception [69], stereopsis [70], and egocentric spatial representation in the dorsal pathway. Activation of the postcentral gyrus may also be linked to the feeling of presence and vividness or interactivity experienced by an individual in a 3D sketching environment [71]. These regions emphasize the complexity of 3D sketching processes and their connection to visual–spatial cognition functions. Contrary to our expectations, across all conditions, the PPA and RSC did not have a significant impact on the 3D sketching environment compared to the 2D environment. According to previous findings [72], we considered that this may be related to the fact that our sketching space setting did not provide enough scene features and elements.

Given the immersive and navigational aspects of 3D sketching tools, they have great potential to enhance spatial experiences and sensory engagement in 3D environments. Our study answers the research question by demonstrating that viewing sketching processes in 2D and 3D environments results in different visual and spatial brain activations. Moreover, our study reaffirms that different sketching objects in a 3D sketching setting demand higher visual and spatial cognition. These findings emphasize the role of 3D spatial sketching tools and highlight their ability to activate specific neural processes.

Furthermore, our findings offer novel insights into the impact of a 3D tool on creative spatial thinking, exemplified by a comparison to freehand sketching. Previous literature has discussed how sketching can be used as a thinking tool to externalize concepts, explore ideas, and solve problems [73]. We further extend our horizon to the concept of “R-mode thinking,” which refers to a creative, holistic, and intuitive way of thinking [74]. When individuals sketch in a 3D sketching environment, they may enhance their ability to perceive and understand the spatial relationship between immersion and the feeling of presence on a different scale. When tapping into their “R-mode thinking,” it is clear that the thinking focus shifts away from precise details and more toward visual and spatial perceptions. This finding implies that the “R-mode” system of thinking plays a significant role when individuals work with 3D sketching tools. Future research could aim to establish behavioral correlations with neural activity to explore how sketching in a 3D environment might elicit R-mode thinking during sketching processes, thus affecting spatial perception and creativity in design.

While our study provides valuable insights, it has limitations that require acknowledgment. To enhance the applicability of our findings, future research should use upgraded hardware and diverse experimental designs, including behavioral data. Additionally, our study mainly focused on landscape design students. Expanding the research to untrained individuals or other academic disciplines can broaden its relevance. Future research should also delve into the intricate relationship between brain activity and behavior, especially in the context of spatial thinking, to reveal practical implications. Furthermore, exploring the potential of immersive VR technologies for creating lifelike representations of complex structures holds promise for future studies in this field.

**Fig 8:**
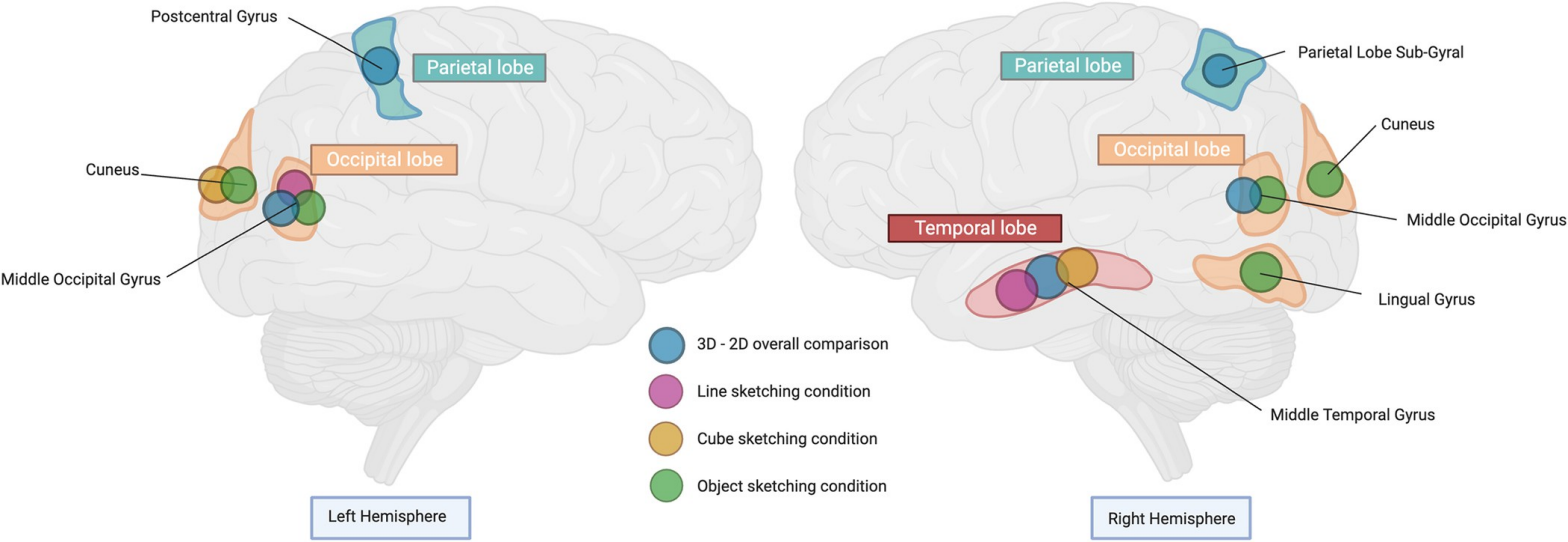
Differences in brain activation when viewing 2D and 3D sketching environments. Illustration publication number: IN2620T6E1.

In conclusion, our study underscores the cognitive advantages associated with the use of 3D VR sketching tools, which elicit enhanced visual and spatial brain activation compared to traditional 2D tools. These insights hold substantial promise for advancing design thinking and have implications for future developments in spatial thinking and VR-based design sketching. Furthermore, our study provides neural evidence of brain activation during 3D sketching, as depicted in Fig 8. Moving forward, it is imperative for future research to establish links between this neural activity and behavioral outcomes, thereby facilitating a more comprehensive understanding of the impact of 3D sketching on cognitive processes.
